# Distribution pattern of Tugai forests species diversity and their relationship to environmental factors in an arid area of China

**DOI:** 10.1371/journal.pone.0232907

**Published:** 2020-05-13

**Authors:** Yong Zeng, Chengyi Zhao, Zbigniew W. Kundzewicz, Guanghui Lv

**Affiliations:** 1 State Key Laboratory of Desert and Oasis Ecology, Xinjiang Institute of Ecology and Geography, Chinese Academy of Sciences, Urumqi, Xinjiang, China; 2 College of Resources and Environmental Sciences, Xinjiang University, Urumqi, Xinjiang, China; 3 University of Chinese Academy of Sciences, Beijing, China; 4 Land Science Research Center, Nanjing University of Information Science & Technology, Nanjing, China; 5 Institute of Agricultural and Forest Environment, Polish Academy of Sciences, Poznan, Poland; Shandong University, CHINA

## Abstract

Ecological restoration of degraded riparian Tugai forests is a key driver to combat desertification in arid regions. Previous studies have focused mainly on changes in groundwater as the underlying mechanisms of Tugai forest’s decline. We evaluated species composition and diversity of Tugai forest and their relationship to groundwater, soil salinity, and soil nutrient. Using 73 quadrats (100 m × 100 m) from 13 transects located perpendicularly to river in the upper reaches of the Tarim River. Eighteen plant species belonging to sixteen genera and eight families were recorded, and the dominant species included *Populus euphratica*, *Phragmites communis*, and *Tamarix ramosissima*. Three *P*. *euphratica* stand ages were detected: young stand, mature stand, and old stand. There were significant differences in species diversity, groundwater depth, groundwater salinity, distance from the quadrat to the river channel, soil moisture content, pH, electrical conductivity, total salt, Cl^−^, SO_4_^2−^, Ca^2−^, Mg^2+^, Na^+^, K^+^, soil organic carbon, and soil organic matter across the stand ages. Seven species were identified as indicators of the three stand ages. Redundancy analysis indicated that the Tugai forest diversity indices were negatively correlated with groundwater depth, groundwater salinity, and distance from the river, and positively associated with electrical conductivity, total salt, pH, Cl^−^, SO_4_^2−^, CO_3_^2−^, soil organic matter, soil organic carbon, and soil moisture content. Plant diversity was the highest at 3–6 m groundwater depth, followed by 0–3 m and then 6–9 m, with the lowest recorded at > 9 m. The appropriate groundwater depth for herbs was about 1–4 m, whereas the depth for trees and shrubs was about 3–6 m. The groundwater depth < 6 m was deemed suitable for the growth of desert riparian forests. This results provide a scientific reference for the ecological restoration and protection for Tugai forests in arid areas.

## Introduction

Desertification is the serious land degradation at arid, semi-arid and dry sub-humid areas [1], which constitute the largest terrestrial ecosystem, collectively covering 41% of the Earth’s land surface and supporting over 38% of the human population [2]. More than 70% of all drylands are affected by desertification [3]. Desertification leads serious ecological consequence such as soil properties deterioration and biodiversity loss [4]. Vegetation degradation is a major contributory factor to desertification [5].

Tugai is a form of riparian vegetation and forest that occurs along large rivers in Central Asian deserts, such as the Tarim River, Syr Darya, and Amu Darya [6]. Tugai forests almost exclusively consist of the tree species *P*. *euphratica* [7]. Among these areas, the largest natural *P*. *euphratica* forest is in the Tarim Basin of China, which accounts for > 50% of the global *P*. *euphratica* forests [8]. As a natural barrier to desert expansion, these forests provide valuable ecosystem services, including biodiversity maintenance, protection from sandstorms, moderation of desertification, regulation of oasis climate, forest soil fertilization, maintenance of ecosystem balance, and the most importantly, they prevent desertification in arid regions [8, 9]. However, due to a lack of water resources, the habitat conditions of Tugai forests have changed [10]. The area of Tugai forest has reduced from 5.4 × 10^4^ hm^2^ in the 1950s to 0.67 × 10^4^ hm^2^ in the 1990s [11]. Tugai forest degradation is often accompanied by large changes in the spatial pattern of soil resources and vegetation [12], which have been linked with alterations in the structure and functioning of ecosystem ultimately leading to its desertification [13]. Therefore, determining the species diversity of Tugai forests is crucial for Tugai forest restoration and combat desertification in arid regions [7].

To date, numerous studies have reported on *P*. *euphratica* in Asia, Europe, and North Africa. These studies mainly focused on stress resistance [14], structural characteristics [7, 9], morphological characteristics [15], physiological characteristics [11], quantitative characteristics [16], and water utilization strategies [17]. Previous studies have shown that changes in groundwater depth can strongly affect the growth of *P*. *euphratica* [6, 18, 19]. With increased groundwater depth, the tree cover and density become sparser [19]. Due to *P*. *euphratica* absorbed and utilized limited water through the soil, soil moisture, salinity and nutrient were considered to be primary eco-environmental factors in Tugai forests ecosystem [20, 21]. Soil moisture and nutrient availability were certified to be effective in explaining plant diversity [22, 23, 24]. For example, soil organic matter can reflect changes in plant species diversity in Tarim River [22]. The seedlings regeneration of *P*. *euphratica* was positively correlation with topsoil salinity in Heihe River [25]. Therefore, groundwater depth, soil moisture, soil nutrient and soil salinity likely determine Tugai forests species diversity. The Tarim River is a 1321-km-long inland river located in the Tarim Basin, which is the most arid basin in China [8]. More than 90% of the Tugai forest area consists of *P*. *euphratica* [9]. The growth and maintenance of Tugai forests are highly dependent on groundwater availability [26]. Prior studies have indicated that declines in the groundwater table are detrimental to Tugai vegetation [27]. Furthermore, soil salt and nutrients also impact plant growth and development in riparian forests [22]. For example, *P*. *euphratica* have “nurse effect” and “fertile island effect”, which can concentrate soil organic matter, soil salt under canopies, and contribute to the survival of herb plants [28, 29]. Soil under tree canopy have significantly higher contents of soil organic matter and soil salt than those in open space [28]. The enrichment of “fertile island” is different across *P*. *euphratica* age and were ranked increasing order as: small *P*. *euphratica*, medium *P*. *euphratica* and big *P*. *euphratica* [29]. Former studies have mainly report on *P*. *euphratica* and few studies reported on the shrub and herb undergrowth in *P*. *euphratica* stands [18, 19]. In fact, these two functional plant types also play important roles in maintaining the stability of Tugai forests [30]. Therefore, different plant functional types should be considered for protecting and managing Tugai forests [7, 21].

Our objectives were to (1) characterize and compare Tugai forest composition and diversity across stand age; and (2) quantify the effects of environmental factor on the species diversity of Tugai forests. This study is expected to provide a theoretical basis and scientific guidance for Tugai forest protection and restoration.

## Material and methods

### Study area

The Tarim River, with an area of 17,600 km^2^, is located in the Tarim Basin. The mean annual temperature is 10.6–11.5°C, mean sunshine duration is 2729.0 h, and the total solar radiation is 5796 MJm^−2^a^−1^ [27]. This region is an extremely arid region, with a mean annual precipitation of 50–70 mm and mean annual evaporation of 2100–3000 mm [31]. The tree *P*. *euphratica* is the constructive species, which has absolute advantage in abundance and coverage. The undergrowth plants include *Phragmites communis*, *Tamarix ramosissima*, *T*. *hispida*, *Glycyrrhiza inflate*, *Karelinia caspica*, *Halimodendron halodendron*, *Calamagrostis pseudophragmites*, *Lycium ruthenicum*, and *Alhagi sparsifolia* [24, 26].

### Quadrat surveys

**Fig 1:**
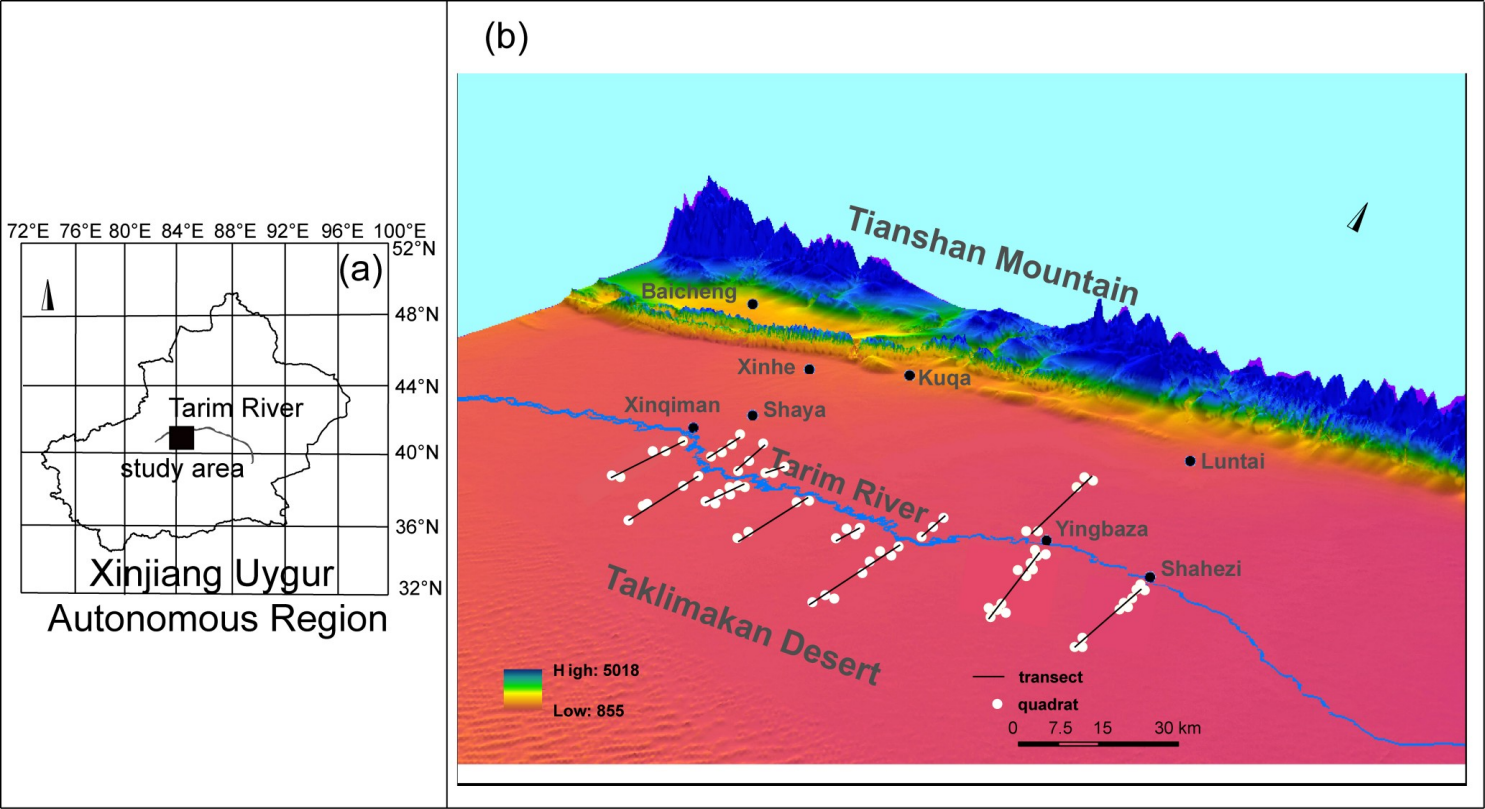
The location of quadrats in upper reaches of Tarim River. (a) The location of study area. (b) 73 quadrats from thirteen transects.

No permission was required to perform the survey because vegetation grow in public area in the upper reaches of the Tarim River. The specific permit for scientific research is not required. To capture the vegetation characteristics in the upper reaches of the Tarim River, 13 transects from Xinqiman to Shahezi were selected in July 2016 (S1 Table). The transect was approximately 1.5–30 km in length. The distance between adjacent quadrats ranged from 0.5 km to 9 km on a vertical channel. There were three to twelve quadrats (100 m × 100 m) in each transect. A total of 73 quadrats (100 m × 100 m) from 13 transects were established (Fig 1). The quadrat was divided into sub-quadrats (25 m × 25 m) to survey the numbers, canopy widths, and heights of the shrubs and trees. The diameter of the trees at breast height (DBH) (breast height = 1.3 m) was calculated for each tree (≥ 5 cm DBH) and sapling (DBH < 5 cm). In the sub-quadrat, four sampling quadrats (size 5 m × 5 m) were established to survey herb heights, coverage, and density [32].

### Stand age of *P*. *euphratica*

To determine the stand age, the relationship between *P*. *euphratica* age and DBH was adopted rather than increment coring. Xu et al. [33] studied the relationship between age and DBH by measuring DBH of *P*.*euphratica* age 5 to 137 year and determined the stand age of the trees using *y* = 4.086 + 0.3956 × *x* + 0.0286 × *x*^2^ (adjusted *R*^2^ = 0.999, *p* < 0.0001), in which *y* is stand age and *x* is DBH [34]. All stands were classified into three stand age classes: young (< 20 years), mature (21–75 years), and old stands (> 75 years) [19]. There were 19 plots in the young stands, 30 plots in the mature stands, and 24 plots in the old stands.

### Environmental factors

In each quadrat, soil samples were collected at a depth of 0–20 cm. Each soil sample was composed of soil from five different locations. A conductivity meter was used to determine electrical conductivity [35]. A glass electrode pH meter was used to determine soil pH [36]. The dry residue method was used to determine total salt [37]. AgNO_3_ titration and ethylenediaminetetraacetic acid (EDTA) indirect titration were used to determine chloride (Cl^−^) and sulfate (SO_4_^2−^), respectively [37]. The double indicator neutral method was used to test carbonate (CO_3_^2−^) and bicarbonate (HCO_3_^−^) [37]. The flare photometer method was used to determine sodium (Na^+^) and potassium (K^+^) [37]. The oven-drying method was used to determine soil moisture content [21]. The oil bath-K_2_CrO_7_ titration method was used to determine soil organic matter (SOM) and soil organic carbon (SOC) [20]. Groundwater salinity (GS) was determined based on the method reported in Zhou [38]. Ground penetrating radar (Italy RIS-2K) and nuclear magnetic resonance (NMR developed by Vista Clara Company) were used to determine groundwater depth [21]. The location of the quadrat was recorded using a GPS.

### Statistical analysis

#### Plant importance value and diversity

Importance value, density, frequency, and cover were considered to be important indices for determining the dominant species of the plant community [39]. The species importance value index was calculated according to the following:

Importance value (IV) = relative density + relative frequency + relative cover.

The species diversity of the Tugai forest was determined using the simple diversity index (*D*), Shannon–Wiener index (*H*), Hill’s diversity index (*H*_*a*_), and Pielou’s evenness index (*E*). The following formulae were used:

$$D=1-\sum P_{i}{}^{2}$$ (1)

$$H=-\sum P_{i}\text{ln}P_{i}$$ (2)

$$H_{a}=(\sum_{i=1}^{S}p_{i}^{a}{)}^{1/1-a}$$ (3)

E=H/lnS (4)

where *P*_*i*_ is the proportion of the *i*th species, and ln is the natural logarithm [40]. *S* is the total number of species [41]. The value of *a* is variable value across the range 0–2. When *a* = 0, *H*_*a*_ = *S*; *a* = 1, *H*_*a*_ = *e*^*H*^; *a* = 1, *H*_*a*_ = 1/D.

#### Indicator species

To calculate an indicator value, the abundance and fidelity of species to a particular community were used [21, 42] according to the following:

$$RA_{mj}=Y_{mj}/\sum_{m=1}^{g}Y_{mj}$$ (5)

$$Y_{mj}=\sum_{i=1}^{nm}a_{ijm}/n_{m}$$ (6)

$$RF_{mj}=\sum_{i=1}^{nm}b_{ijm}/n_{m}$$ (7)

$$b_{ij}=a_{ij}^{0}$$ (8)

$$IV_{mj}=100(RA_{mj}\times RF_{mj})$$ (9)

where *IV*_*mj*_ is the *j*th species in the community *m*, *RA*_*mj*_ is the abundance of the *j*th species in the community *m*, *RF*_*mj*_ is the fidelity of the *j*th species in the community *m*, *a*_*ijm*_ refers the abundance of the *j*th species in the *i*th quadrat of the community *m*, *n*_*m*_ refers the total number of quadrats in community *m*, *g* refers to the number of the communities, *a*_*ij*_ is the matrix of quadrat × species and is transformed to a presence-absence. The *IV*_*mj*_ values ranged from 0 to 100. The 0 and 100 represent not present and perfect indication. The *IV*_*mj*_ value > 25 indicates that species have significant indicator values.

#### Relationship between plant diversity and environmental factors

In our study, environmental factors were analyzed, such as distance from quadrat to river channel, groundwater depth, groundwater salinity, soil moisture content, soil salt and nutrient, and impact on plant species diversity, using redundancy analysis (RDA) [21]. RDA was calculated using CANOCO (Version 4.5). Figures were drawn with CanoDraw (Version 4.0).

Gaussian regression was used to investigate the relationship between plant species and groundwater depth [43, 44]. The following formulae were used:

$$f(x)=\frac{1}{\sqrt{2\pi \sigma x}}e^{-\frac{1}{2}{\left(\frac{\text{ln}x-\mu }{\sigma }\right)}^{2}}$$ (10)

$$X_{pm}=e^{u-\sigma^{2}}$$ (11)

$$E(X)=e^{u+\frac{1}{2}\sigma^{2}}$$ (12)

$$\sigma (X)=e^{u+\frac{1}{2}\sigma^{2}}{\left(e^{\sigma^{2}}-1\right)}^{\frac{1}{2}}$$ (13)

where *x* is groundwater depth, and the mathematical expectation and standard deviation of ln*x* are *u* and *σ*, respectively. *X*_*pm*_ is the mode of appearance frequency of a plant, which indicates the appropriate groundwater depth. *E*(*X*) is the mathematical expectation of groundwater depth. *σ*(*X*) is the standard deviation of groundwater depth.

## Results

### Plant composition in the Tugai forest

We identified eighteen species across the seventy-three quadrats, including sixteen genera from eight families (Table 1). The families included Chenopodiaceae (four species, four genera), Leguminosae (four species, four genera), Tamaricaceae (three species, one genus), Salicaceae (one species, one genus), Compositae (four species, four genera), Solanaceae (one species, one genus), Apocynaceae (one species, one genus), and Gramineae (two species, two genera). Of the 18 species, 50% and 44.4% of the total were shrubs and herbs. The dominant plants were *P*. *euphratica*, *T*. *ramosissima*, and *P*. *communis*, and the associated importance value indices were 158.8%, 102.5%, and 86.3%, respectively.

**Table 1:** Family, genera, functional types of 18 plant species and their relative density (RD%), relative frequency (RF%), relative cover (RC%) and important value index (IVI%) at 73 quadrats.

Family	Genera	Species	Life forms	RD%	RF%	RC%	IVI%
Salicaceae	Populus	1 *Populus euphratica*	tree	7.74	100.00	51.08	158.82
Tamaricaceae	Tamarix	2 *Tamarix ramosissima*	shrub	7.98	75.34	19.19	102.51
		3 *T*. *hispida*	shrub	0.76	13.70	1.37	15.84
		4 *T*. *arceuthoides*	shrub	0.23	17.81	0.74	18.78
*Chenopodiaceae*	*Halostachys*	5 *Halostachys caspica*	shrub	4.05	21.92	9.81	35.78
	*Halocnemum*	6 *Halocnemum strobilaceum*	shrub	0.28	9.59	0.05	9.92
	*Halogeton*	7 *Halogeton glomeratus*	herb	5.43	36.99	0.30	42.72
	*Chenopodiaceae*	8 *Salsola ruthenica*	herb	2.97	20.55	0.22	23.73
Compositae	Karelinia	9 *Karelinia caspica*	herb	0.83	16.44	0.79	18.06
	Hexinia	10 *Hexinia polydichotama*	herb	0.09	4.11	0.01	4.21
Leguminosae	Halimodendron	11 *Halimodendron halodendron*	shrub	0.07	2.74	0.04	2.85
	Alhagi	12 *Alhagi sparsifolia*	shrub	4.49	2.74	1.14	8.79
	Glycyrrhiza	13 *Glycyrrhiza inflata*	herb	8.81	0.11	3.74	12.66
	Sophora	14 *Sophora alopecuroides*	herb	0.05	1.37	0.05	1.47
Solanaceae	Lycium	15 *Lycium ruthenicum*	shrub	1.33	10.96	1.51	13.80
Apocynaceae	Apocynum	16 *Poacynum henderson*	shrub	4.91	2.74	1.14	8.79
Gramineae	Phragmites	17 *Phragmites communis*	herb	48.72	30.14	7.43	86.28
	Calamagrostis	18 *Calamagrostis pseudophragmites*	herb	0.55	1.37	0.22	2.14

### Plant diversity across *P*. *euphratica* stand ages

**Fig 2:**
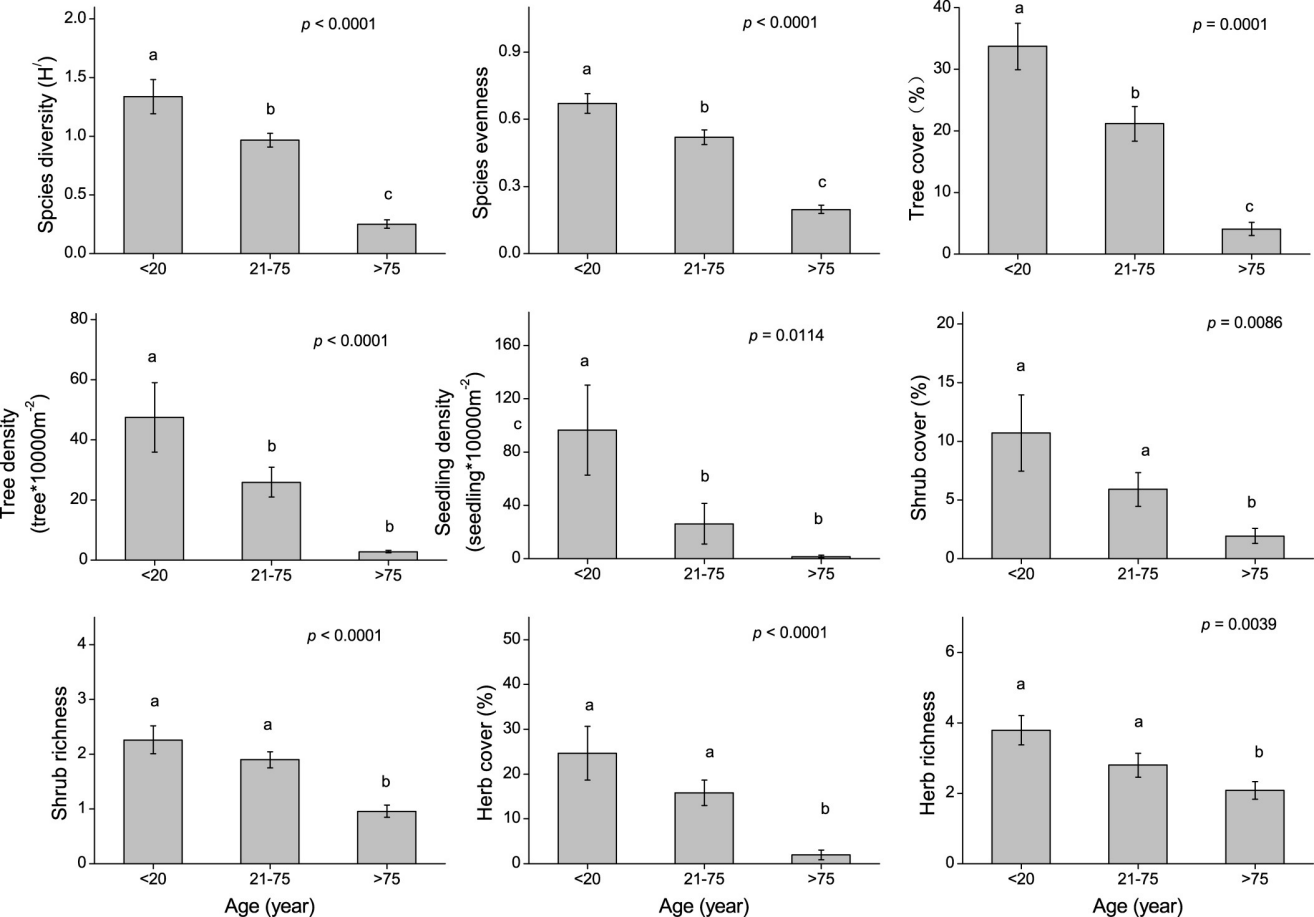
Changes in plant species diversity across *P*. *euphratica* stand ages.

Species diversity, evenness, seedling density, tree cover and density, and shrub and herb cover and richness differed across the stand ages. Species diversity, evenness, seedling density, tree cover and density, and shrub and herb cover and richness were highest in the young stands, followed by the mature stands and then the old stands (Fig 2). Seven species were identified as indicators of the three ages classes (Table 2). The old stands had two indicator species, the mature stands had three indicator species, and the young stands had two indicator species.

**Table 2:** Indicator species associated with young (Y), mature (M), and old stand (O) age classes.

Species		Y versus M			Y versus O			M versus O		
Shrubs		Y	M	*P*	Y	O	*P*	M	O	*P*
*T*. *ramosissima*	M	24.7	22.2	0.174	36.4	9.8	0.190	**40.4**	12.1	0.002
*L*. *ruthenicum*	M	6.9	50.3	0.012	39.4	6.1	0.789	**54.1**	1.1	0.011
*H*. *strobilaceum*	Y	**29.4**	14.7	0.042	35.0	11.6	0.080	24.2	25.2	0.715
Herbs										
*P*. *communis*	Y	**27.4**	21.9	0.037	45.3	0	0.063	42.6	0	0.816
*K*. *caspica*	M	9.6	**46.1**	0.032	34.2	12.6	0.783	**50.7**	3.9	0.029
*S*. *ruthenica*	O	33.8	7.8	0.998	1.5	**53.9**	0.035	0.28	**44.2**	0.019
*H*. *glomeratus*	O	0.0	61.2	0.732	0.0	**55.8**	0.047	31.9	18.9	0.740

For comparison, the data shown are the *P*. *euphratica* stand indicator value in each age and *P* value for each large indicator value. Strong habitat indicator species (shown in bold) have indicator value > 25 and *p* < 0.05.

### Environmental factors associated with *P*. *euphratica* stand age

Groundwater depth, groundwater salinity, and distance from quadrat to river channel were significantly different across stand age (Table 3). Groundwater depth, groundwater salinity, and distance from quadrat to river channel were highest in the old stands, following by the mature stands and then the young stands. Soil moisture content, pH, electrical conductivity, total salt, Cl^−^, SO_4_^2−^, Ca^2−^, Mg^2+^, Na^+^, K^+^, soil organic carbon, and soil organic matter differed significantly across stand age and were ranked in decreasing order as young stand, mature stand, and old stand. Altitude was ranked in decreasing order as young stand, old stand, and mature stand.

**Table 3:** Mean value (±SE) of environmental factors across young (Y), mature (M), and old stand (O) age classes.

Environmental factors	Y	M	O
Groundwater depth (m)	3.7 (±0.4) c	7.5 (±0.6) b	16.9 (±0.5) a
Groundwater salinity (g/L)	1.5 (±0.1) c	4.6 (±0.2) b	7.8 (±0.3) a
Distance from quadrat to river channel (km)	1.5 (±0.4) c	8.1 (±1.3) b	28.2 (±0.3) a
Soil moisture content (%)	28.2 (±0.3) a	6.0 (±1.4) b	1.1 (±0.2) c
Altitude (m)	957.3 (±5.9) a	938.3 (±4.7) a	955.5 (±4.7) a
pH	8.5 (±0.1) a	8.4 (±0.1) a	8.4 (±0.1) a
Electrical conductivity (ms/cm)	6.1 (±1.1) a	3.9 (±0.6) a	1.7 (±0.2) b
Total salt (g/kg)	24.0 (±4.8) a	14.0 (±2.3) a	6.5 (±1.0) b
CO_3_^2−^ (g/kg)w	0.0 (±0.0) a	0.0 (±0.0) a	0.0 (±0.0) a
HCO_3_^−^ (g/kg)	0.2 (±0.0) a	0.2 (±0.0) a	0.2 (±0.0) a
Cl^−^ (g/kg)	7.7 (±1.4) a	4.5 (±1.0) a	1.8 (±0.3) b
SO_4_^2−^ (g/kg)	8.4 (±1.5) a	5.0 (±0.7) b	1.8 (±0.4) c
Ca^2−^ (g/kg)	1.4 (±0.3) a	1.3 (±0.2) a	0.5 (±0.2) b
Mg^2+^ (g/kg)	0.6 (±0.2) a	0.2 (±0.0) b	0.1 (±0.0) b
Na^+^ (g/kg)	3.6 (±1.0) a	2.3 (±0.5) a	1.5 (±0.2) a
K^+^ (g/kg)	0.3 (±0.0) a	0.3 (±0.0) a	0.1 (±0.0) b
Soil organic carbon (g/kg)	3.8 (±0.5) a	3.2 (±0.3) a	2.0 (±0.2) b
Soil organic matter (g/kg)	7.1 (±1.0) a	5.4 (±0.6) b	3.4 (±0.4) b

### Relationship between environmental factors and species diversity

**Fig 3:**
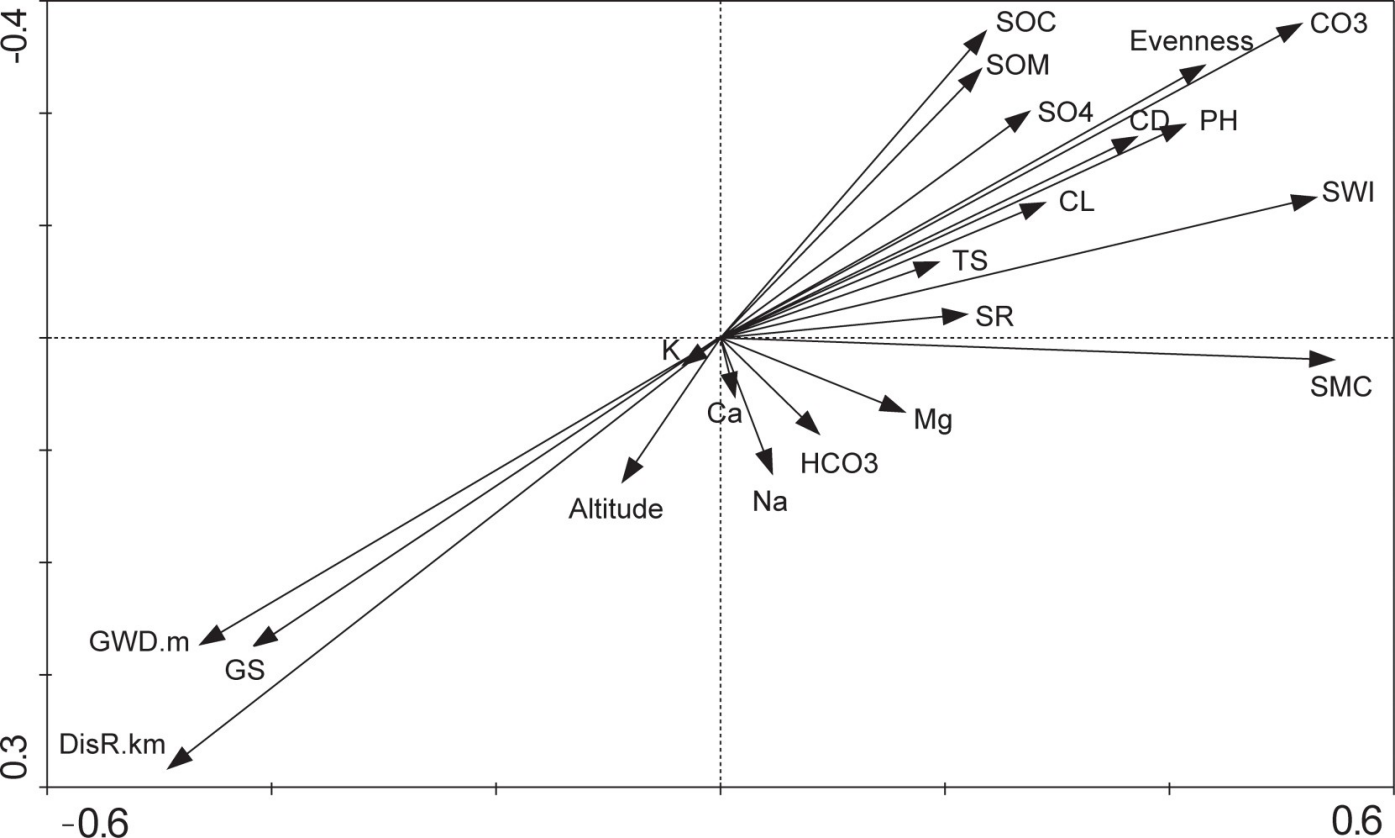
Redundancy analysis of environmental factors impact on plant species diversity. DistR, distance from quadrat to river channel; GWD, groundwater depth; GS, groundwater salinity; SMC, soil moisture content; SOM, soil organic matter; SOC, soil organic carbon; TS, Total salt; EC, electrical conductivity; CO_3_^2−^, carbonate; HCO_3_^−^, bicarbonate; Cl^−^, chloride; SO_4_^2−^, sulfate; Ca^2+^, calcium; Mg^2+^, magnesium; Na^+^, sodium; K^+^, potassium; SR, species richness; SWI, Shannon-Wiener index.

**Fig 4:**
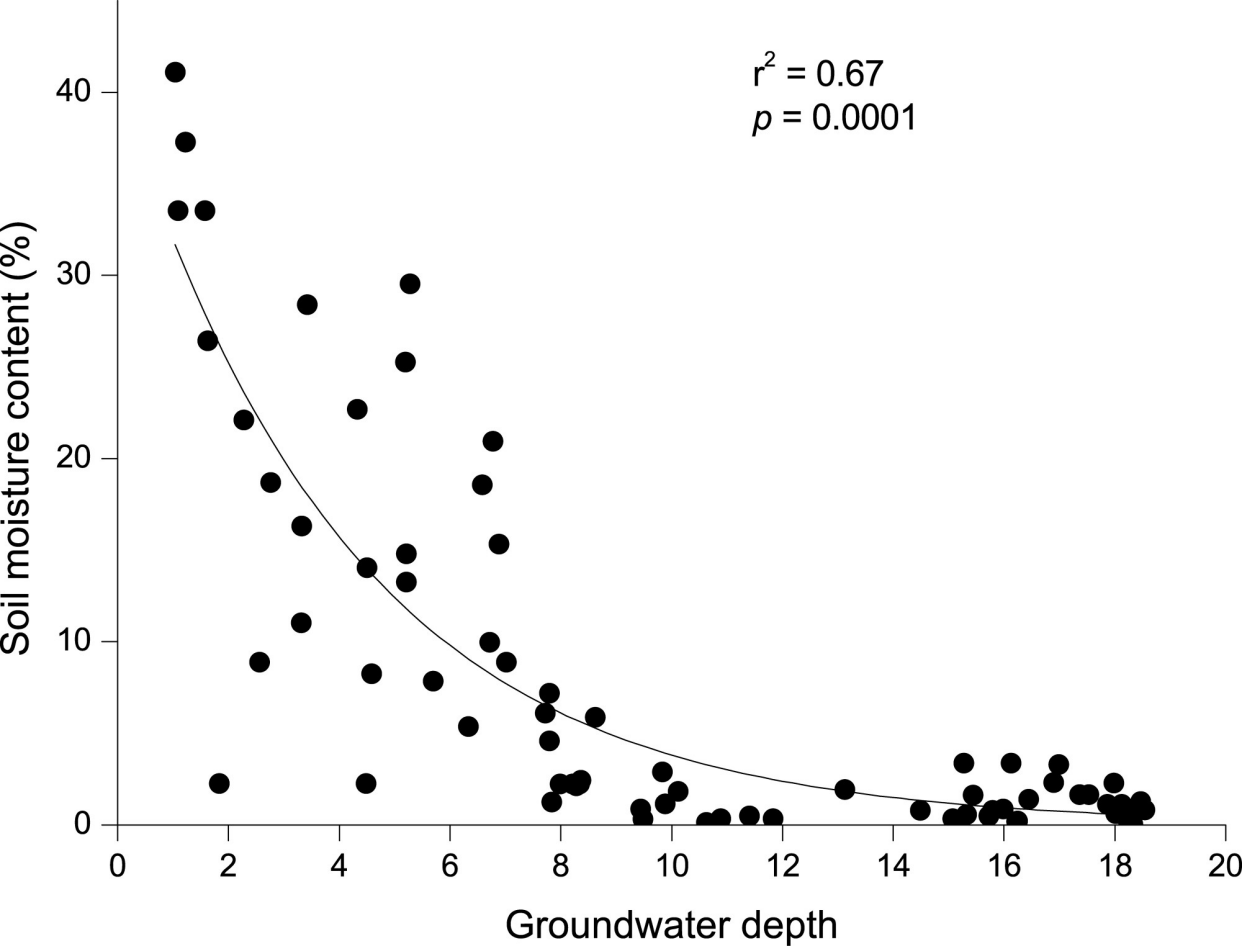
Change in soil moisture content in relation to groundwater depth variable.

**Fig 5:**
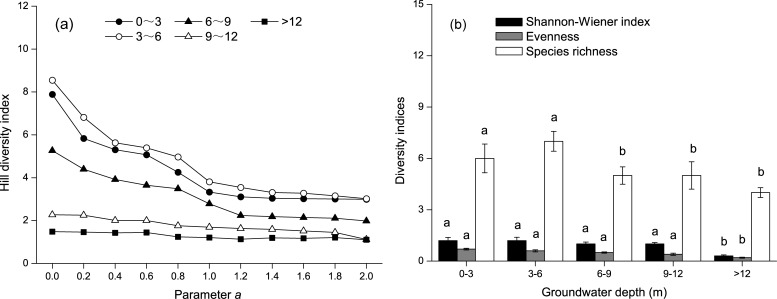
Plant diversity ordination (Hill’s diversity index, Shannon-Wiener index, evenness, species richness) along groundwater depth classes.

**Fig 6:**
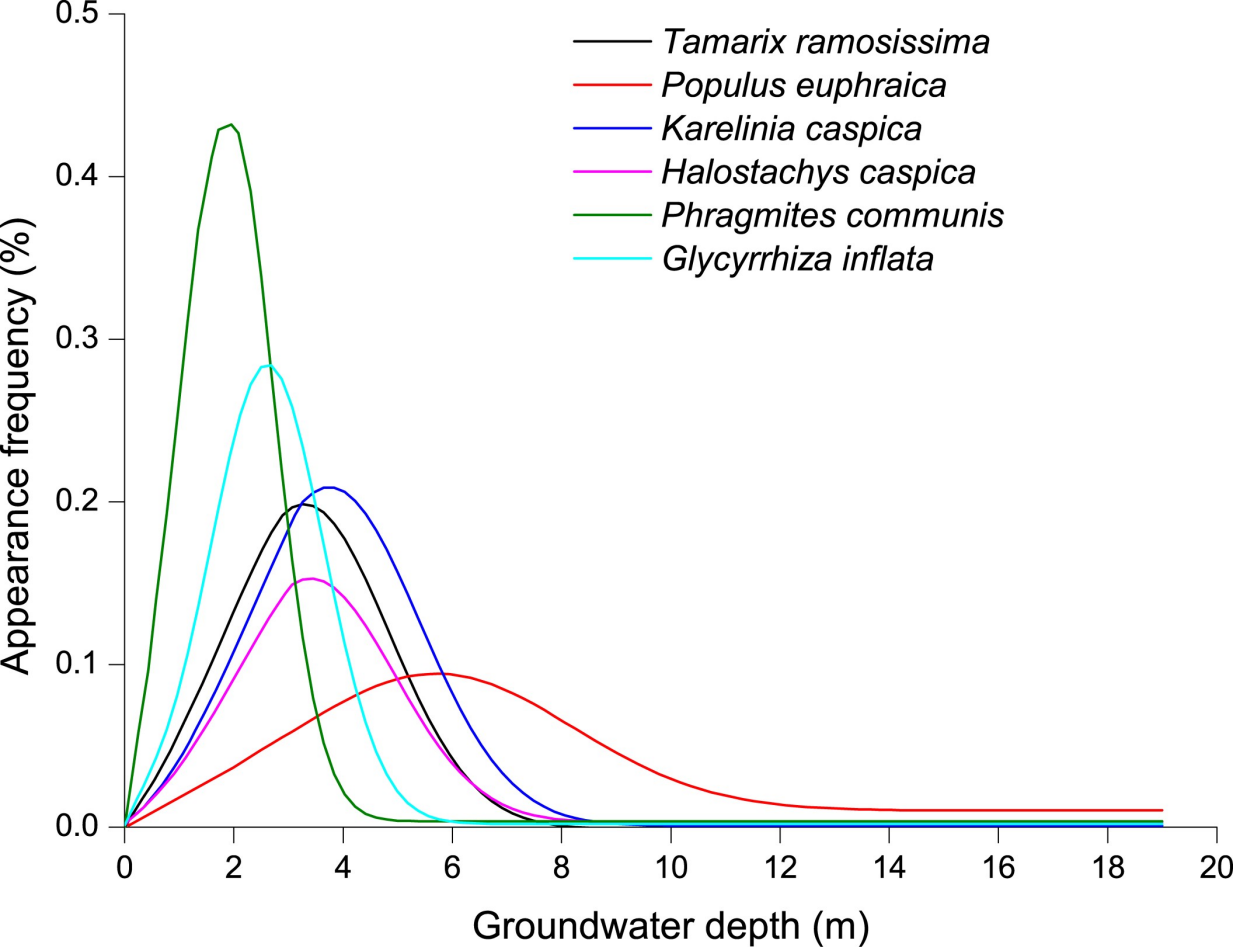
Change in plant in relation to groundwater depth variable.

RDA analysis was used to examine the relationship between environmental factors and species diversity (Fig 3). The first two axes explained 98.5% of the variation. The species-environment correlation was 0.77 in the first axis and 0.66 in the second axis. Plant species richness, the Shannon-Wiener index, and evenness were negatively correlated with both groundwater depth, groundwater salinity, and distance from the river, but were positively correlated with electrical conductivity, total salt, pH, Cl^−^, SO_4_^2−^, CO_3_^2−^, soil organic matter, soil organic carbon, and soil moisture content (Fig 3 and Table 4). Soil moisture content declined with increased groundwater depth (Fig 4). Multiple linear regression analysis indicated that multicollinearity among groundwater depth, groundwater salinity, and distance from the river is strong (*VIF* > 1). The groundwater depth is the most crucial factor for species diversity (*p* < 0.00; Table 5). Hill diversity index, Shannon-Wiener index, and species richness declined in an order corresponding to the groundwater depth of 3–6 m, 0–3 m, 6–9 m, 9–12 m, and > 12 m (Fig 5). Evenness declined in an order corresponding to the groundwater depth of 0–3 m, 3–6 m, 6–9 m, 9–12 m, and > 12 m. In the Gaussian regression analysis between the appearance frequency of 18 plants and groundwater depth, only *P*. *communis*, *T*. *ramosissima*, *P*. *euphratica*, *K*. *caspica*, *Halostachys caspica*, and *Glycyrrhiz inflata* could be analyzed, and appropriate groundwater depths (*X*_*pm*_) corresponding to the peak values of appearance frequency were 1.4 m, 3.0 m, 5.3 m, 3.6 m, 3.3 m, and 2.4 m, respectively (Fig 6 and Table 6). The above data indicated that the appropriate groundwater depth for herbs was about 1–4 m, whereas the depth for trees and shrubs was about 3–6 m.

**Table 4:** Pearson correlation coefficients between plant diversity and environmental factors.

Evironmental factors	species richness	Shannon-Wiener index	Evenness
Groundwater depth (m)	−0.544**	−0.857**	−0.830**
Groundwater salinity (g/L)	−0.523 **	−0.661**	−0.752**
Distance from quadrat to river channel (km)	−0.545**	−0.755**	−0.748**
Soil moisture content (%)	0.472**	0.789**	0.829**
Altitude (m)	0.05	0.206	0.202
pH	0.267*	0.224*	0.235*
Electrical conductivity (ms/cm)	0.305**	0.533**	0.636**
Total salt (g/kg)	0.214*	0.520**	0.611**
CO_3_^2−^ (g/kg)	0.360*	0.394**	0.388**
HCO_3_^−^ (g/kg)	0.078	0.114	0.036
Cl^−^ (g/kg)	0.283*	0.532**	0.620**
SO_4_^2−^ (g/kg)	0.339**	0.541**	0.635**
Ca^2−^ (g/kg)	0.060	0.251*	0.330**
Mg^2+^ (g/kg)	0.131	0.361**	0.398**
Na^+^ (g/kg)	0.000	0.364**	0.424**
K^+^ (g/kg)	0.094	0.359**	0.398**
Soil organic carbon (g/kg)	0.252*	0.408**	0.440**
Soil organic matter (g/kg)	0.242*	0.379**	0.418**

**Table 5:** Multiple liner regression analysis results of species diversity (stepwise selection method).

Model	Regression coefficients *B*	*t*	*p* value	Adjust *R*^*2*^	*F*	*F* statistic *p* value	Collinearity statistics	
							Tolerance	*VIF*
Constant	1.52	14.75	0.00					
Groundwater depth (m)	−0.07	−7.73	0.00	0.45	59.75	0.00	1.00	1.00
Distance from quadrat to river channel (km)	−0.07	−0.25	0.80				0.09	10.71
groundwater salinity (g/L)	−0.30	−1.71	0.09				0.24	4.14

**Table 6:** Parameters of logarithm normal distribution fitting curves of plant species.

Species	*u*	*σ*	*X*_*pm*_	*E*(*X*)	*σ*(*X*)
*Tamarix ramosissima*	1.24	0.38	2.99	3.71	1.46
*Populus euphratica*	2.02	0.59	5.32	8.97	5.78
*Karelinia caspica*	1.45	0.41	3.60	4.63	1.98
*Halostachys caspica*	1.37	0.40	3.35	4.36	1.78
*Phragmites communis*	0.70	0.61	1.39	2.43	1.63
*Glycyrrhiza inflata*	1.01	0.35	2.43	2.91	1.05

## Discussion

### Tugai forests community pattern

Eighteen plant species belonging to eight families and sixteen genera were detected in the upper reaches of the Tarim River, which is similar to that of the Syr Darya and Amu Drya rivers [45], which harbor large areas of Tugai forest [7]. Although poor in species richness, Tugai forests act as a sand stabilizer in arid desert regions [18]. Our results indicated that the importance value of *P*. *euphratica* in the Tarim River was the highest, followed by *T*. *ramosissima* and *P*. *communis*. This differs from the riparian forest in southwestern USA, where *Tamarix* has established stands and excludes native *Populus* species [46]. Therefore, *P*. *euphratica* is the unique species responsible for formulating the forest community in arid desert regions in China [18].

There was considerable variability in species diversity among the different stands, which were ranked in decreasing order as young stands, mature stands, and old stands. The decrease in species diversity from young to old stands is mainly due to the disappearance of herb plants [47]. The groundwater depth was shallow in the young stands, followed by the mature stands and then the old stands (Table 3). Herb species possess shallow roots and can thus easily access water in shallow groundwater areas [48]. In deeper groundwater, the deep-rooted tree *P*. *euphratica* will provide water to the surrounding herb species, but plants growing far from *P*. *euphratica* will die from lack of water [10]. Therefore, herb cover and richness in the young stands were significantly higher than in the old stands, which corroborates the research of Soykan et al. [30] conducted in the San Pedro River in southeastern Arizona.

Seven indicator species were detected among the young, mature, and old stands. The indicators of the young stands, *P*. *communis* and *Halocnemum strobilaceum*, grow at shallow groundwater depths near the riverside and exhibit phenotypic plasticity, and are thus more adaptable in saline soil [49]. Two indicators of mature stands, *T*. *ramosissima* and *L*. *ruthenicum*, are deep-rooted shrubs [50] that are salt-tolerant and drought-tolerant [51]. The indicators of old stands, *Halogeton glomeratus* and *Salsola ruthenica*, are annual herbs. These herbs were positively associated with *P*. *euphratica*, which provides soil salt, nutrients, a sheltering microhabitat and reduces the surface temperature of the soil in the summer [50]. Therefore, *P*. *euphratica* is a “nurse plant” and have “fertile island effect” for these two herbs.

### The effects of environmental factors on plant diversity

Different age structures of the stands reflect continuous periods of regeneration [19]. Our findings indicated that *P*. *euphratica* stand age differed significantly across a transverse gradient (i.e., perpendicular to the channel), where young stands are near to the channel and old stands are near to the desert margin. This may be because the average groundwater depth of the riverside habitat is 3.7 m and the average value of distance from quadrat to river channel is about 1.5km in the young stands. Tugai forests grow better in the riverside habitat than in other areas [52]. This may be the seed bank activation and regeneration of *P*. *euphratica* requires floods disturbance [23]. The vitality of *P*. *euphratica* highly depends on the shallow groundwater depth [53]. For example, the newly generated branches of *P*. *euphratica* and the number, length, width, and weight of 50 leaves of those newly generated branches fall with increasing distance from the river channel [31]. In the old stands, the average groundwater depth of the desert margin habitat is 16.9 m. Gries et al. [54] and Thomas et al. [24] found that *P*. *euphratica* tolerates groundwater depth beyond 20 m. However, deep groundwater depth (≥ 14.0 m) is considered to be unsuitable for the growth of Tugai forest [55]. Therefore, most trees have perished and the seedling density is low in the old stands [56].

In this study, soil salinity was not the limiting factor for *P*. *euphratica* forests. This may be because the seedling regeneration of *P*. *euphratica* was positively associated with topsoil salinity [50]. In addition, *P*. *euphratica*, *T*. *ramosissima*, *P*. *communis*, and *K*. *caspica* withstand high salinity because they have evolved biochemical, physiological, anatomical, and molecular mechanisms to tolerate salinity stress [23, 49]. For example, *P*. *euphratica* has a high capacity to exclude NaCl ions and can regenerate under high salinity [57], while *T*. *ramosissima*, *P*. *communis*, and *K*. *caspica* can remove Na^+^ ions from the cytoplasm efficiently and maintain the required K^+^ concentration [49]. Our study implied that groundwater depth, groundwater salinity, and distance from quadrat to river channel are crucial driving forces for *P*. *euphratica* forest degradation. With greater groundwater depth and the increase in groundwater salinity and distance from quadrat to river channel, plant species richness, Shannon-Wiener Index, and evenness declined (Fig 3). This is consistent with the conclusion of Naumburg et al. [48] and Li et al. [11], who found that increased groundwater depth obstructed community structure and intensified desertification.

Groundwater depth is the most crucial controlling factor for Tugai forest species diversity in our arid study area (Table 5). A suit depth of groundwater determined is significant to ensure the stability of a desert riparian forest ecosystem [58]. Thus, numerous studies have reported appropriate groundwater depths based on the ecological parameters of vegetation and the ecological responses to water stress in desert riparian forests [44, 59]. In the Daly River of Australia, riparian plants can grow normally at groundwater depths < 5m [60]. In the Heihe River of Northwest China, the appropriate groundwater depth should not exceed 5–6 m to support the growth of desert riparian vegetation [59]. In our study, Hill diversity index, Shannon-Wiener index, and species richness were the highest at 3–6 m groundwater depth, followed by 0–3 m and then 6–9 m, with the lowest recorded at > 9 m. In 0–3 m groundwater depth area, the ground surface is remarkably flat [61]. Ecological niche overlap was phenomenal among the trees, shrubs and herbs [61]. Plant species diversity was higher at a groundwater depth < 6 m, and species diversity declined significantly when the groundwater depth was deeper than 6 m. This indicated that a groundwater depth < 6 m was suitable for maintaining higher species diversity of desert riparian forests in the Tarim River. This finding is also supported by Hao et al. [44] and Keyimu et al. [62], which have shown that species diversity is high at a groundwater depth < 6 m.

In the past 40 years, The Tarim River suffered from vegetation decline and desertification [63]. For example, a total of 1.23 × 10^4^ km^2^ of land was affected by desertification [64]. The area of shrub and meadow vegetation decreased by 200km^2^ [64]. Ecological restoration of degraded riparian Tugai forests is a key driver to combat desertification [13]. In arid areas, the tree-shrub-herb structures is highly stable and have a stronger sand stabilization ability than tree structure [65]. In our study, the appropriate groundwater depths of *P*. *euphratica* was 5.3 m. This finding is supported by Fan et al. [66] and Chen et al. [67], who found that the suitable range of depths to the groundwater table to maintain the growth of *P*.*euphratica* was previously reported to range from 4 to 9m. The appropriate groundwater depth for herbs was about 1–4 m, whereas the depth for trees and shrubs was about 3–6 m, indicated that the groundwater depth that permitted restoration of herb plants was 1–4 m, trees and shrubs was about 3–6 m. Therefore, we suggest that to protect the riparian plant community, different plant functional types, rather than some, should be considered for conservation. Conservation managers need to ensure that a sufficient amount of plant functional types is maintained for the structural and functional sustainability of the riparian forest. This finding has great significance for the restoration and protection of damaged desert riparian ecosystems.

## Supporting information

S1 Table (XLSX)

S1 Data (DOCX)
