# Supplementary material for: Distribution pattern of Tugai forests species diversity and their relationship to environmental factors in an arid area of China
Source: PLoS One. 2020 May 13;15(5):e0232907. doi: 10.1371/journal.pone.0232907 (PMC7219784; doi:10.1371/journal.pone.0232907)
Supplement: S1 Data — (DOCX) [file pone.0232907.s002.docx]

**Distribution pattern of** **Tugai forests species diversity and** **their relationship to environmental factors in an arid area of China**

Yong Zeng^1,2,3^, Chengyi Zhao^4^*, Zbigniew W. Kundzewicz^4,5^, Guanghui Lv^2^

^1^ *State Key Laboratory of Desert and Oasis Ecology,* *Xinjiang Institute of Ecology and Geography, Chinese Academy of Sciences, Urumqi 830011, Xinjiang, China*

^2^*College of Resources and Environmental Sciences, Xinjiang University, Urumqi 830046, Xinjiang, China*

^3^*University of Chinese Academy of Sciences, Beijing 100049, China*

*^4^ Land Science Research Center,* *Nanjing University of Information Science & Technology, Nanjing 210044, China*

*^5^Institute of Agricultural and Forest Environment, Polish Academy of Sciences, Poznan 61-819, Poland*

*Corresponding author

E-mail: [zhaocy@nuist.edu.cn](mailto:zhaocy@nuist.edu.cn)

These authors contributed equally to this work.

**Table A1 The geographic coordinates of 13 transects**

|  |  | Geographic coordinate | |  |  | Geographic coordinate | |
| --- | --- | --- | --- | --- | --- | --- | --- |
| Transect 1 | Quadrat 1 | N 41°0′48″ | E 82°47′12″ | Transect 2 | Quadrat 5 | N 40°46′49″ | E 83°20′2″ |
|  | Quadrat 2 | N 41°0′54″ | E 82°47′40″ |  | Quadrat 6 | N 40°58′4″ | E 83°24′1″ |
|  | Quadrat 3 | N 41°1′6″ | E 82°48′15″ |  | Quadrat 7 | N 40°53′7″ | E 83°22′13″ |
|  | Quadrat 4 | N 41°0′50″ | E 82°47′19″ |  | Quadrat8 | N 40°51′23″ | E 83°21′14″ |
| Transect 3 | Quadrat 9 | N 41°0′14″ | E 82°49′16″ | Transect 4 | Quadrat12 | N 40°57′59″ | E 82°54′55″ |
|  | Quadrat 10 | N 41°0′2″ | E 82°48′32″ |  | Quadrat13 | N 40°57′22″ | E 82°54′57″ |
|  | Quadrat11 | N 41°0′42″ | E 82°50′53″ |  | Quadrat14 | N 40°57′9″ | E 82°54′42″ |
| Transect 5 | Quadrat 15 | N 41°10′52″ | E 84°13′37″ | Transect 6 | Quadrat20 | N 40°44′20″ | E 81°49′4″ |
|  | Quadrat 16 | N 41°11′53″ | E 84°14′54″ |  | Quadrat21 | N 40°43′12″ | E 81°48′36″ |
|  | Quadrat 17 | N 41°18′32″ | E 84°11′18″ |  | Quadrat22 | N 40°39′45″ | E 81°45′59″ |
|  | Quadrat18 | N 41°19′36″ | E 84°12′10″ |  | Quadrat23 | N 40°34′32″ | E 81°50′42″ |
|  | Quadrat19 | N 41°19′0″ | E 84°11′23″ |  | Quadrat24 | N 40°34′33″ | E 81°51′45″ |
| Transect 7 | Quadrat25 | N 40°56′5″ | E 83°48′21″ | Transect 8 | Quadrat32 | N 40°54′52″ | E 83°9′30″ |
|  | Quadrat26 | N 40°56′27″ | E 83°49′3″ |  | Quadrat33 | N 40°55′44″ | E 83°10′7″ |
|  | Quadrat27 | N 40°56′57″ | E 83°49′44″ |  | Quadrat34 | N 40°54′31″ | E 83°10′16″ |
|  | Quadrat28 | N 40°46′2″ | E 83°37′17″ |  | Quadrat35 | N 40°55′52″ | E 83°10′51″ |
|  | Quadrat29 | N 40°47′31″ | E 83°40′13″ |  | Quadrat36 | N 40°54′12″ | E 83°8′40″ |
|  | Quadrat30 | N 40°47′59″ | E 83°38′42″ |  | Quadrat37 | N 40°54′16″ | E 83°8′55″ |
|  | Quadrat 31 | N 40°57′11″ | E 83°49′44″ |  | Quadrat 38 | N 40°54′29″ | E 83°8′45″ |
| Transect 9 | Quadrat39 | N 40°54′35″ | E 83°4′1″ | Transect 10 | Quadrat44 | N 40°59′31″ | E 83°28′40″ |
|  | Quadrat40 | N 40°54′24″ | E 83°3′54″ |  | Quadrat45 | N 40°59′30″ | E 83°28′37″ |
|  | Quadrat41 | N 40°46′34″ | E 82°42′47″ |  | Quadrat46 | N 40°59′38″ | E 83°28′41″ |
|  | Quadrat 42 | N 40°45′59″ | E 82°41′54″ |  | Quadrat47 | N 40°59′35″ | E 83°28′36″ |
|  | Quadrat 43 | N 40°47′12″ | E 82°45′1″ |  |  |  |  |
| Transect 11 | Quadrat48 | N 41°10′3″ | E 84°18′58″ | Transect 12 | Quadrat60 | N 41°9′49″ | E 84°43′5″ |
|  | Quadrat49 | N 41°9′51″ | E 84°19′27″ |  | Quadrat61 | N 41°8′26″ | E 84°38′47″ |
|  | Quadrat50 | N 41°6′23″ | E 84°17′11″ |  | Quadrat62 | N 41°10′29″ | E 84°44′2″ |
|  | Quadrat51 | N 41°7′20″ | E 84°17′45″ |  | Quadrat63 | N 41°10′22″ | E 84°43′45″ |
|  | Quadrat52 | N 41°8′41″ | E 84°21′20″ |  | Quadrat64 | N 41°10′16″ | E 84°43′43″ |
|  | Quadrat53 | N 40°58′20″ | E 84°16′9″ |  | Quadrat 65 | N 41°10′8″ | E 84°43′20″ |
|  | Quadrat 54 | N 40°57′50″ | E 84°16′0″ |  | Quadrat66 | N 41°9′33″ | E 84°42′31″ |
|  | Quadrat55 | N 40°56′56″ | E 84°15′40″ |  | Quadrat67 | N 41°6′54″ | E 84°33′7″ |
|  | Quadrat56 | N 40°58′1″ | E 84°17′42″ |  | Quadrat68 | N 41°0′17″ | E 84°28′21″ |
|  | Quadrat57 | N 41°0′26″ | E 84°14′0″ |  | Quadrat69 | N 41°59′56″ | E 84°29′52″ |
|  | Quadrat 58 | N 41°11′32″ | E 84°20′53″ |  | Quadrat70 | N 41°1′1″ | E 84°30′2″ |
|  | Quadrat 59 | N 41°11′29″ | E 84°20′10″ |  |  |  |  |
| Transect 13 | Quadrat71 | N 41°4′47″ | E 83°59′39″ |  |  |  |  |
|  | Quadrat 72 | N 41°6′31″ | E 83°59′7″ |  |  |  |  |
|  | Quadrat 73 | N 40°5′7″ | E 83°59′23″ |  |  |  |  |
